# Chemical and Biological Evaluation of Essential Oils from Cardamom Species

**DOI:** 10.3390/molecules23112818

**Published:** 2018-10-30

**Authors:** Emira Noumi, Mejdi Snoussi, Mousa M. Alreshidi, Punchappady-Devasya Rekha, Kanekar Saptami, Lucia Caputo, Laura De Martino, Lucéia Fatima Souza, Kamel Msaada, Emilia Mancini, Guido Flamini, Abdulbasit Al-sieni, Vincenzo De Feo

**Affiliations:** 1 Laboratory of Bioressources: Integrative Biology & Recovery, High Institute of Biotechnology, University of Monastir, Monastir 5000, Tunisia; emira_noumi@yahoo.fr; 2 Department of Biology, College of Science, University of Ha′il, Hai’l 2440, Saudi Arabia; snmejdi@yahoo.fr (M.S.); mousa.algladi@gmail.com (M.M.A.); 3 Laboratory of Genetics, Biodiversity and Valorisation of Bioresources, High Institute of Biotechnology, University of Monastir 5000, Tunisia; 4 Yenepoya Research Centre, Yenepoya University, Mangalore 575018, India; rekhapd@hotmail.com (P.-D.R.); kanekarsaptami@gmail.com (K.S.); 5 Department of Pharmacy, University of Salerno, Via Giovanni Paolo II, 132, I-84084 Fisciano (Salerno), Italy; luciaa.caputoo@gmail.com (L.C.); luceia.souza@ufrgs.br (L.F.S.); emancini@unisa.it (E.M.); defeo@unisa.it (V.D.F.); 6 Laboratory of Medicinal and Aromatic Plants, Biotechnology Center in Borj-Cedria Technopole, BP. 901, Hammam-Lif 2050, Tunisia; msaada_kamel@hotmail.com; 7 Department of Pharmacy, University of Pisa, via Bonanno, 6, 56126 Pisa, Italy; guido.flamini@farm.unipi.it; 8 Department of Biochemistry, Faculty of Science, King Abdul Aziz University, Jeddah 21589, Saudi Arabia; aalsieni@kau.edu.sa

**Keywords:** green cardamom, Ethiopian cardamom, black cardamom, chemical composition, antimicrobial activity, allelopathic activity

## Abstract

To highlight the importance of the spices in the Mediterranean diet, the aim of the paper was to study the essential oil compositions and to clarify the potential differences in the biological activities of the three cardamom species. In the study, we compared the phytochemical profiles and biological activities of essential oils from *Elettaria cardamomum*, *Aframomum corrorima* and *Amomum subulatum*. The oils were analyzed using the GC and GC/MS techniques and were mainly constituted of the oxygenated monoterpenes which represents 71.4%, 63.0%, and 51.0% of all compounds detected in *E. cardamomum*, *A. corrorima* and *A. subulatum* essential oils, respectively, 1,8-cineole was the main common compound between the tree tested volatile oil. The essential oils showed significant antimicrobial activity against Gram-positive and Gram-negative microorganisms tested especially the fungal strains. The Ethiopian cardamom was the most active essential oil with fungal growth inhibition zone ranging from 12.67 to 34.33 mm, MICs values ranging from 0.048 to 0.19 mg/mL, and MBCs values from 0.19 to 1.75 mg/mL. The three tested essential oils and their main component (1,8-cineole) significantly increased the production of elastase and protease production, and motility in *P. aeruginosa* PAO1 in a dose dependent manner. In fact, at 10 mg/mL concentration, the three essential oils showed more than 50% of inhibition of elastolytic and proteolytic activities in *P. aeruginosa* PAO1. The same oils inhibited also the violacein production in *C. violaceum* strain. It was also noticed that at high concentrations, the *A. corrorima* essential oil significantly inhibited the germination of radish. A thorough knowledge of the biological and safety profiles of essential oils can produce applications of economic importance.

## 1. Introduction

Aromatic and medicinal plants offer a wide range of bioactive molecules used to contrary the spread of drug resistance pathogenic bacteria and fungi that cause severe and life-threatening infections [1,2]. Spices and aromatic plants are commonly used in Mediterranean regions in food preparation due to their antibacterial activities against several pathogenic Gram positive and Gram negative bacteria including: *Listeria*, *Vibrio*, *Staphylococcus*, *Pseudomonas*, *Salmonella*, Bacillus and *Microccocus* genera with multidrug resistance profiles [3,4]. The increasing of multi-resistance developed in most community, and hospital acquired pathogens can be attributed to the high use of antibiotics by consumers to feat pathogens. The scientific community developed since many years an interest in aromatic and medicinal plants with antimicrobial properties [1].

Essential oils are aromatic oily liquids with very complex nature known for their antimicrobial and medicinal properties [1]. Nowadays, some volatile oils and their mains components are used in food industry as antimicrobials due to their ability to control natural spoilage food process and to prevent growth of foodborne pathogens acting as food preservation and food safety molecules [3].

It is well known that both Gram positive and Gram negative pathogenic bacteria regulate their virulence traits using the quorum sensing (QS) system throughout the expression of a variety of genes as function of bacterial cell density [5]. For example, many virulence properties are regulated by the QS system including the synthesis of violacein in *Chromobacterium violaceum* ATCC 12472 [6,7], biofilm formation, swarming on semi-solid agar medium, production of elastase, protease and pyocyanin in the *Pseudomonas aeruginosa* PAO1 [8]. Several aromatic/medicinal essential oil and organic extract have been tested for their anti-QS activities [9,10]. A large variety of plant-derived molecules were described for their ability to interfere with the autoinducers molecules synthesized by Gram positive bacteria (AIP) and Gram negative bacteria (AHL) and attenuates the behavior of the QS-controlled virulence factors expression in both *C. violaceum* and *P. aeruginosa* PAO1 biomonitor strains [11,12].

The cardamom of commerce is called small green cardamom or the true cardamom (*Elettaria cardamomum*). There are many other plants belonging to the *Amomum* and *Aframomum* genera, both belonging to the cardamom family, producing aromatic seeds. Among them, clubbed together as false cardamoms, the most important and the one that is being grown commercially is *Amomum subulatum*. Most of these false cardamoms are used as flavoring plants and also as remedies for various ailments [13]. *Elettaria cardamomum* (L.) Maton is a tall, perennial, reed-like herb growing wild in rainforests of South India, Sri Lanka, and other tropical countries [9,10]. The plant is one of the world very ancient and expensive spices and is known as “the queen of spices” [14]. *Amomum subulatum* (A. Braun) P.C.M. Jensen, black cardamom or large cardamom [15], is native to Sikkim. The Sikkim State of India alone contributes 50% of the world production of large cardamom [16]. Korarima (*Aframomum corrorima* (Braun) P.C.M. Jansen), also called Ethiopian cardamom or false cardamom, is native to Ethiopia but it is cultivated on a small scale in some West African countries. Korarima is a perennial, aromatic herb, and bearing flowers either terminally on aerial leaf shoots or from the ground level. Korarima is one of the aromatic medicinal plants used in traditional medicine by the people of southern Ethiopia [17,18,19].

Specifically, Eyob and colleagues [18] conducted an ethnobotanical survey in the southern regions of Ethiopia on the three major korarima growing wild including Gamo Gofa, Debub Omo and Kaffa. They identified 38 and 52 compounds, respectively in leaf and rhizome essential oils. The major component of the oil of the leaf was β-caryophyllene (60.7%). The rhizome oil was dominated by γ-terpinene (21.8%) and β-pinene (17.6%).

It has been reported that in the chemical composition of different species of cardamoms, monoterpenes are the main compounds [14,17,20,21,22]. Also, antimicrobial activity of the cardamoms is reported [23,24]. On black cardamom, 1,8-cineole is the main component [15,25]. Eyob and co-workers [17,18] reported the essential oil composition of Ethiopian cardamom. The presence of different structures of biomolecules in the volatile oils, allows a wider spectrum of bioactivity. Recently, it has been demonstrated that organic extracts from *Amommum* tsao-ko (Crevost et Lemarie) and *Elettaria cardamomum* exhibited anti-quorum sensing and antibiofilm activities [26,27].

The aims of this paper was to compare the different essential oil composition and to clarify the potential differences in the biological activities of the three tested species (including antimicrobial, allelopathic, and anti-quorum activities) as they are considered by plant sellers as the same “cardamom” species.

## 2. Results

### 2.1. Essential Oil Composition

Components of the essential oils, obtained by hydrodistillation and analyzed by gas chromatography-mass spectrometry (GC-MS) technique, are divided into five class compounds based on their chemical functional groups (Table 1).

The major class compounds were the oxygenated monoterpenes which represents 71.4%, 63.0%, and 51.0% of all compounds detected in *E. cardamomum*, *A. corrorima* and *A. subulatum* essential oils, respectively. This family is dominated by 1,8-cineole where its maximum percentage was observed in the green cardamom (55.4%), followed by the Ethiopian cardamom (51.8) and the black cardamom (41.7).

The monoterpene hydrocarbonss were the second dominant components in the three cardamom varieties and represent 36.9% in the green cardamom essential oil, followed respectively by the black cardamom (34.2%) and the Ethiopian cardamom (24.7%). The second class of components is dominated by α-terpinyl acetate (28.6%) in green cardamom essential oil, 4-terpineol for the Ethiopian cardamom (10.4%), and geraniol (12.5%) in the essential oil of black cardamom.

The sesquiterpenes hydrocarbons were found only in the essential oil of the *A. corriroma* and *A. subulatum* representing (0.5%) and (0.6%) of the total compounds identified, respectively. The oxygenated sesquiterpenes were also found in the black and Ethiopian cardamom essential oils representing 1.6% and 1.3% of the total compounds identified, respectively.

**Figure 1:**
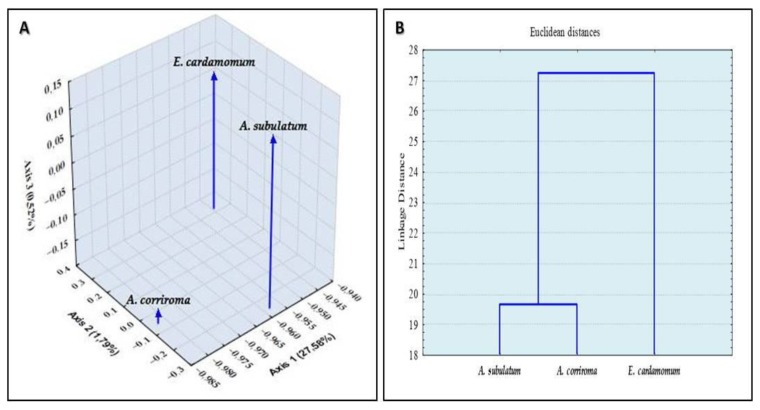
(**A**) Relative positions of the three essential oils tested based on their phytochemical composition in the space defined by the three principal components. (**B**) Cluster analysis obtained in the cluster analysis of the essential oils of green cardamom, Ethiopian cardamom, and black cardamom based on the data (Table 1): horizontal, samples analyzed; vertical, differentiation level between the three essential oils tested.

The principal component analysis (PCA) was carried out in order to determine the relationship between the three cardamom varieties on the basis of their essential oil composition. A better discrimination was revealed on the three dimensional visualization of the plotted scores. Results obtained from the PCA showed the existence of three well-defined groups clearly distinguished both in quality and in quantity (Figure 1A).

These results confirm that, despite the presence of the 1,8-cineole as a main component in the three essential oils, they are both qualitatively and quantitatively chemically different. Results obtained from the cluster analysis (Figure 1B) confirmed the existence of one well-defined group represented by the black and Ethiopian cardamom oils suggesting similar compositions. The green cardamom essential oil was clearly distinguished from the latter group both in quality and in quantity.

### 2.2. Antimicrobial Activities

The antibacterial activity of the three essential oils was tested using the disc diffusion assay and using the microdilution assay on twenty five Gram-positive and Gram-negative bacteria including those frequently associated with food contamination and human disorders. The results were recorded as the diameters (mm) of the growth inhibition zone and as a minimal inhibition and bactericidal concentrations (mg/mL) (Table 2 and Table 3).

For the antifungal activity (Table 2), the results showed that the three essential oils were also active against the yeast strains tested with high diameters of fungal growth inhibition zone ranging from 14.33 to 21.67 mm for the green cardamom oil, from 12.67 to 34.33 mm for the Ethiopian cardamom oil, and from 6.0 to 40.33 mm for the black cardamom oil. The Ethiopian cardamom was the most active essential oil with MICs values ranging from 0.048 to 0.19 mg/mL, and MBCs values from 0.19 to 1.75 mg/mL. Using the Duncan’s test comparing the mean diameters of the microbial growth inhibition zones, independently of the microorganism tested, Corriroma essential oil was classified as the most active oil (mean diameter 17.75 mm) followed by the green cardamom oil (mean diameter 16.27 mm) and finally the Ethiopian cardamom oil (mean diameter 16.21 mm).

The essential oils showed significant antimicrobial activity against all Gram-positive and Gram-negative microorganisms tested, giving inhibition zones ranging between 6 and 41.33 mm for the green cardamom essential oil, between 11.33 and 32.00 mm for the Ethiopian cardamom essential oil, and between 6 to 43 mm for the black cardamom essential oil. The high diameters of the growth inhibition zones were obtained when Gram-positive bacteria are tested for the three volatile oils.

Indeed, the highest activity was observed on *S. epidermidis* ATCC 12228 (diameter 43.00 mm) for the black cardamom essential oil, on *M. luteus* NCIMB 8166 (diameter 41.33 mm) for the green cardamom essential oil, and on *S. aureus* ATCC 6816 (diameter 32.00 mm) for the Ethiopian cardamom oil.

Using the microdilution assay, the results showed that weak concentrations of the three essential oils tested were sufficient to inhibit the growth of all microorganisms tested (MICs values), and to completely stop the bacterial growth (MBCs values). In fact, the lowest MICs values were ranged from 0.048 to 0.19 mg/mL for Ethiopian cardamom oil, and from 0.048 to 0.097 mg/mL for the green and black cardamom oils. Concentrations as low as 0.39 mg/mL was sufficient to reproduce a bacteriostatic effect for the black Ethiopian cardamom oil, 0.78 mg/mL for the black cardamom oil, and 6.25 mg/mL for the green cardamom oil (Table 3).

The results of the effects of the three essential oils and their main component 1,8-cineole on virulence factors production in *P. aeruginosa* PAO1 showed an inhibition of the motility in a concentration dependent manner (Table 4).

Under control conditions, PAO1 strain give a diameter about 54 mm and in the presence of different concentrations of the three essential oils and their main component (1,8-cineole), the bacteria were able to grow and form a colony in the center with diameter not exceeding 8.33 mm. In fact, the highest level of inhibition in the migration of PAO1 was recorded with the green and ethiopian volatile oils at 400 µg/mL. The diameter of the colony ranged from 54 ± 1 mm to 8.33 ± 0.58. 1,8-Cineole was also able to inhibit the swarming ability of the PAO1 strain with a diameter ranging from 17.33 ± 1.15 mm at 10 µg/mL to 10.33 ± 0.58 mm at 400 µg/mL.

**Figure 2:**
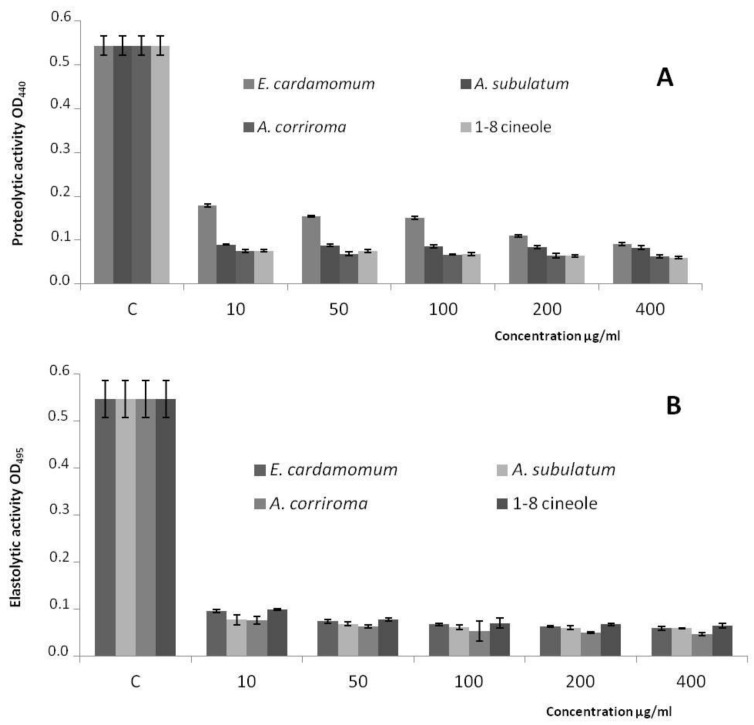
The effect of the tested essential oils and 1,8-cineole on Quorum Sensing-regulated proteolytic (**A**) and Quorum Sensing-regulated elastolytic (**B**) activities in P. aeruginosa PAO1. Values are expressed as mean ± SD.

The tested essential oils and their main component (1,8-cineole) decreased the production of the QS-controlled virulence factors (elastase and protease) in *P. aeruginosa* PAO1 in a concentration dependant manner. At 10 µg/mL concentration, the three essential oils showed more than 50% of inhibition of elastolytic and proteolytic activities in *P. aeruginosa* PAO1 (Figure 2A,B).

**Figure 3:**
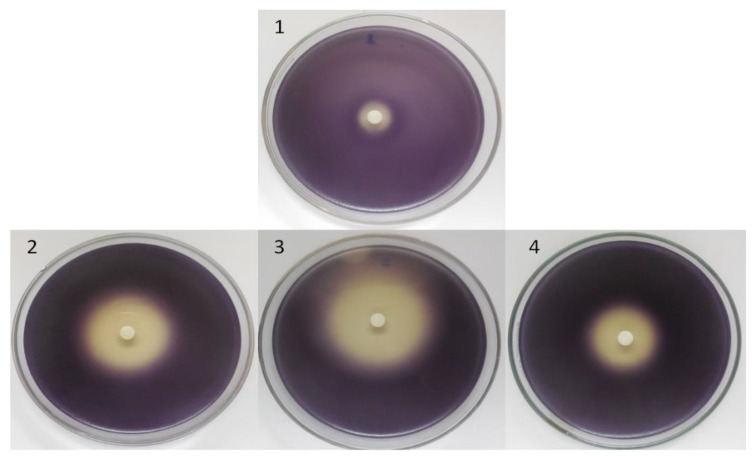
Anti-QS activity of 1,8-cineole (**1**) Ethiopian cardamom oil (**2**) green cardamom oil (**3**) and black cardamom oil (**4**) as demonstrated by the inhibition of C6-Acyl Homoserine Lactone-mediated violacein production in *C. violaceum* 026 starter strain.

The three tested essential oils and their main component (1,8-cineole) exhibited anti-QS activity against *C. violaceum* 026 starter strain. After 24 h incubation, the weakest clear turbid halo zone of 14 mm diameter of violacein inhibition was recorded for the black cardamom and 1,8-cineole at the concentration of 2 mg/disc. The highest diameter of violacein inhibition was noted for the green cardamom about 28 mm and 16 mm with the Ethiopian cardamom essential oil (Figure 3).

### 2.3. Phytotoxic Activity

In this study, the three essential oils were evaluated for their activity against germination and radicle elongation of radish (*Raphanus sativus* L.), garden cress (*Lepidium sativum* L.), and lettuce (*Lactuca sativa* L.).

Doses of 2.5, 1.25, and 0.625 µg/mL of *A. corrorima* essential oil inhibited significantly the germination of radish, but not influenced the germination of the other species. Instead, *A. subulatum* and *E. cardamomum* essential oils not influenced the germination of the species considered (Table 5). None of three oils, instead, seem to be effective against radicle elongation of all these species (data no shown).

## 3. Discussion

Our results showed that the tested *A. subulatum* essential oil was characterized by high proportion of monoterpenes (84.6) dominated by the 1,8-cineole and geraniol. Really, in all essential oils tested, 1,8-cineole was the dominant compound found with a percentage varying from 41.7 to 55.4%. The obtained results were in agreement with those reported in the literature. In fact, in a previous work, we reported that the green cardamom was particularly rich in oxygenated monoterpenes (88.7%) with a dominance of α-terpinyl acetate (45.6%), 1,8-cineole (26%). Additionally, several reports have shown that the basic green cardamom aroma from different geographic origin is a combination of 1,8-cineole and α-terpinyl acetate with different percentages [25]. In fact, Leela and coworkers [28] studied the content and the chemical composition of seven cardamom genotypes from India at different maturity levels (stage of capsule development) and reported that α-terpinyl acetate and 1,8-cineole were always the main components. In 2006, Hymete and colleagues [29] reported that the seed oil of *Aframomum corrorima* from Ethiopia was particularly rich on the monoterpenes 1,8-cineole (44.3%) and sabinene (17.3%), whereas the sesquiterpenic compounds (*E*)-nerolidol (17.2%), β-caryophyllene (9.7%) and caryophyllene oxide (6.9%) dominated the composition of the husk oil. Eyob and colleagues [18] conducted an ethnobotanical survey in the southern regions of Ethiopia on the three major korarima growing wild including Gamo Gofa, Debub Omo and Kaffa. They identified 38 and 52 compounds, respectively in leaf and rhizome essential oils. The major component of the oil of the leaf was β-caryophyllene (60.7%). The rhizome oil was dominated by γ-terpinene (21.8%) and β-pinene (17.6%). Similar results have been reported by Baser and Kürkçüoglu [30]. Kumar and co-workers [19] reported the chemical composition of the hydrodistilled essential oil obtained from the fruits of *A. subulatum* grown in northeast region of Sikkim (India) by using the capillary GC and GC-MS techniques. They reported that the hydrodistillated oil was dominated by 1,8-cineole (65.39%), α-terpineol (10.15%), β-pinene (7.23%). In 2013, Joshi and colleagues [31] reported the GC-MS profile of the essential oils from the different locations in India. The oils collected showed qualitative and quantitative differences in composition with a dominance of oxygenated monoterpenes (65.31–75.54%), monoterpene hydrocarbons (10.53–17.12%), terpene alcohols (15.32–18.80%), and sesquiterpene hydrocarbons (5.02–9.19%). The oxygenated monoterpene 1,8-cineole (50.55–60.46%) represent the major compound in the oils collected from all regions tested.

Our results confirmed the antimicrobial activity of the three essential oils tested independently of the microorganisms used: in fact, Grădinaru and colleagues [32] reported anti-*Staphylococcus aureus* ATCC 25923 activity of the green cardamom oil at a concentration of 6.25 mg/mL. While, the methicillin-resistant clinical isolates of *S. aureus* (MRSA) were most susceptible to 1,8-cineole (MIC = 1.25 mg/mL) as compared to the cardamom oil (MIC = 6.25 mg/mL). Additionally, we have previously reported that the green cardamom essential oil was active against a large panel of Gram-positive bacteria (mean diameter= 21.77 mm), cariogenic bacteria (mean diameter= 19.51 mm) and fungi (mean diameter= 39.5 mm), with MICs values ranging from 0.023 to 0.046 mg/mL for all bacterial and fungal strains tested [25]. Recently, Teneva and coworkers [33] reported that the green cardamom oil (chemotype α-terpinyl acetate 39.03 %, eucalyptol 31.53 %) was active against pathogenic *E. coli* ATCC 25922, *E. coli* ATCC 8739, *Salmonella* sp., *S. aureus* ATCC 6538P, and *P. vulgaris* strains with a diameters of inhibition zones between 8 and 10 mm and MICs values from 60 to >600 ppm. In 2017, Mutlu-Ingok and Karbancioglu-Guler [34] reported that the green cardamom oil (chemotype α-terpinyl acetate 43.4%, eucalyptol 29.2%) was active against *Campylobacter jejuni* and *Campylobacter coli* with a diameter of growth inhibition zone ranging from 24.75 mm to 25.58, respectively. The MICs and MBCs values were low for the two *Campylobacter* strains tested (0.025 µL/mL).

For the black cardamom oil, Satyal and coworkers [35] reported an antifungal activity of the black cardamom oil against the fungus *Aspergillus niger* with a MIC value about 313 μg/mL, but also a good antibacterial activity against *B. cereus*, *S. aureus*, *E. coli*, and *P. aeruginosa* strains (MICs values ranging from 313 to 625 μg/mL). A good antibacterial activity was also noticed by Naveed and coworkers [36] on multi-drug resistant bacteria with MIC values ranging from 2.83 mg/mL against *E. coli* (SS1) strain to 9.4 mg/mL against the uropathogenic *S. aureus* strain. It have been also previously demonstrated that the black cardamom oil extracted from whole fruits exhibited good results against *B. pumilis*, *B. subtilis*, *M. luteus*, *S. aureus*, *S. epidermidis*, *P. aeruginosa*, *E. coli*, *C. albicans*, *A. niger* and *S. cerevisiae* strains [37]. The diameter of inhibition zone ranged from 15 mm (*S. cerevisiae*) to 20 mm (*B. pumilis* and *S. epidermidis*).

The volatile oil extracted from the fruits of Ethiopian cardamom showed promising anti-*C. albicans ATCC* 90028 and anti-*S. aureus* DSM 346 activity, with a diameter of inhibition zone about 35 mm and 25 mm, respectively (Bacha et al., 2016) [9]. They also showed an antimicrobial activity against several Gram-positive and negative bacteria including *E. coli* K12 DSM 498, *B. cereus* ATCC 10987, *B. cereus*, and *P. aeruginosa* DSM 1117 ones. The MICs values ranged from 12.5 mg/mL (*E. coli* K12 DSM 498) to >25 mg/mL (*P. aeruginosa* DSM 1117).

The three essential oils tested exhibited pronounced antimicrobial activity against Gram-positive and Gram-negative bacteria, with the highest diameter of growth inhibition zone recorded for *M. luteus* NCIMB 8166 (41.33 mm) and *S. aureus* ATCC 6816 (32.67 mm) by the green cardamom oil. The Gram-positive bacterium, *B. cereus* was the most sensitive bacteria to the black cardamom oil (35.67 mm). This can be explained to the difference in the structure and composition of the cell wall of bacteria belonging to two groups and the different mechanism of action of the various components in each essential oil tested [38]. Using the two biomonitor strains *C. violaceum* and *P. aeruginosa* PAO1, we founded that all tested essential oils and their main component (1,8-cineole) attenuated the expression of the tested QS-controlled virulence factors (violacein pigment production, elastase and protease production, and motility) in a dose dependent manner. Previous reports have shown that the essential oil extracted by hydrodistillation from the green cardamom (chemotype α-terpinyl acetate/1,8-cineole) had anti-QS properties against the sensor plasmid pJBA132 (*E. coli* strain) with a percentage of inhibition about 31% after 24 h of exposure, and a weak activity using the long-chain sensor plasmid pRK-C12 in *Pseudomonas putida* strain (inhibition 21–22%). This result indicated that the chemical compounds founded in the green cardamom oil can compete with C6-HSL autoinducers molecules [26]. Al-Haidari and colleagues [39] reported that the green cardamom methanolic extract exhibited significant elimination of pyocyanin formation, significantly inhibited twitching and swimming motilities, and biofilm formation in *P. aeruginosa* PA14 strain. It has been recently demonstrated that *Amomum tsao-ko* extract exhibited a high biofilm inhibition when tested against *S. typhimurium* (51.96%), *S. aureus* (47.06%), and *P. aeruginosa* (45.28%) at 4 mg/mL. The same authors demonstrated that this extract can inhibit the violacein production (44.59%) and anti-swarming activity by 4 mg/mL extract on *S. typhimurium* and *S. aureus* [27].

For the phytotoxic activity test, different studies have reported that some essential oils and their components are potent inhibitors of seed germination and retard plant growth [40]. Germination and seedling growth bioassays are important preliminary screening methods to determine phytotoxic potential of plant extracts and compounds [41]. *E. cardamomum*, *A. corriroma* and *A. subulatum* essentials oils have been recognized for their wide range of physiological and pharmacological properties but no studies were carried out on their phytotoxic activities. The difference in phytotoxic activity of the oils could be attribuited to their chemical composition. *A. corrorima* essential oil composition is richer in monoterpene hydrocarbons (71.4%) than other essential oils studied. Our results corroborate with De Martino and coworkers [42] that showed monoterpenes phytotoxic activity as good inhibitory radish seed germination in a dose-dependent way.

In conclusion, this study has determined the chemical structure of *Elettaria cardamomum*, *Aframomum corriroma* and *Amomum subulatum* oils, and showed that these oils were mainly composed of the oxygenated monoeterpens, in particular 1,8-cineole. It is evident that the tested oils have effectively reduced/stopped the bacterial and fungal growth as well as the communication of bacterial cells. It is proposed that cardamom oils especially their extracts may have a potential use in clinical settings for microbial infections.

## 4. Materials and Methods

### 4.1. Plant Material

In this work, three varieties of cardamom (fruits/seeds) were purchased from a local market in Jeddah city (KSA) in 2014 including the green cardamom (*Elattaria cardamomum* L. Manton), Ethiopian cardamom (*Aframomum corrorima*) and black cardamom (*Amomum subulatum*). The plants were identified by Pr. Al-Sieni Abdulbasit and voucher specimens were deposited in the CERTE (Technopark of Borj Cedria, Tunisia). One hundred grams of plant seeds were distilled for three hours with distilled water (500 mL) using a Clevenger-type apparatus [43]. The obtained essential oil was refrigerated in sealed glass vials at 4 °C until analysis.

### 4.2. GC–EIMS Analysis and Identification of the Components

Gas chromatography-electron ionization mass spectrometry GC/EIMS analyses were performed with a Varian CP-3800 gas-chromatograph (Varian, Inc., Palo Alto, CA, USA) equipped with a HP-5 capillary column (30 × 0.25 mm; coating thickness 0.25 μm) and a Varian Saturn 2000 ion trap mass detector (Varian, Inc., CA, USA). Analytical conditions: injector and transfer line temperatures at 220 and 240 °C, respectively; oven temperature was programmed from 60 to 240 °C at 3 °C/min; carrier gas helium at 1 mL/min; injection of 0.2 mL (10% hexane solution); split ratio 1:30. Identification of the constituents was based on comparison of the retention times with those of authentic standards, comparing their Linear Retention Indices relative to the series of *n*-hydrocarbons, and by computer matching against commercial (NIST 98 and ADAMS 95) and home-made library mass spectra built up from pure substances and components of known essential oils and MS literature data [44,45,46,47]. Moreover, the molecular weights of all the identified substances were confirmed by GC/CIMS, using methanol as CI ionizing gas [48]. Analysis was also run by using a fused silica HP Innowax polyethylenglycol capillary column (50 m × 0.20 mm i.d., 0.25 μm film thickness). In both cases, helium was used as carrier gas (1.0 mL/min).

### 4.3. Biological Assays

#### 4.3.1. Antimicrobial Activities

The antimicrobial effect of the three volatile oils was tested against 25 food-borne pathogenic bacteria including 25 Gram-positive and Gram-negative bacteria, and against seven yeasts including six *Candida* spp. strains and one *Saccharomyces cerevisiae* strain by using the disk diffusion assay [49,50]. For the experiments, a loopful of the microorganisms working stocks was enriched on a tube containing 9 mL of enrichment broth (Mueller Hinton for bacteria and Sabouraud Chloramphenicol for yeast) then incubated at 37 °C for 18–24 h. The overnight cultures were used for the antimicrobial activity of the essential oils used in this study and the optical density was adjusted at 0.1 (OD_600nm_). The inoculums were streaked onto MH (1% NaCl for *Vibrio* strains) agar plates using a sterile swab. While, the yeast strains were inoculated onto Sabouraud dextrose agar (Biolife, Milan, Italy) and incubated for 18 h at 37 °C. The yeast cultures were harvested and then suspended in sterile saline (0.8% NaCl) and the cell density was adjusted to 10^7^ cells/mL (OD_540_= 0.5). The fungal inoculums were streaked onto fresh Sabouraud dextrose agar plates using a sterile swab.

A sterile filter discs (diameter 6 mm, Biolife, Milan, Italy) were impregnated with 10 mg of each essential oil. Ampicillin (10 mg/mL) was used in this study as positive controls for all bacterial strains tested. The dishes were incubated at 37 °C for 18–24 h. The diameter of the zones of inhibition around each of the disks was taken as measure of the antimicrobial activity. Each experiment was carried out in triplicate and the mean diameter of the inhibition zone was recorded.

#### 4.3.2. Microdilution Method for the Determination of the MIC, MBC/MFC

The minimal inhibition concentration (MIC) and the minimal bactericidal concentration (MBC) were determined for all bacteria tested in this study as previously described [51,52]. The minimal inhibition concentration (MIC) and the minimal fungicidal concentration (MFC) were determined for all *Candida* strains as described by Noumi and coworkers [7].

The inocula of the bacterial and fungal strains were prepared from 12 h broth cultures and suspensions were adjusted to 10^7^ UFC/mL. The essential oils dissolved in 10% dimethylsulfoxide (DMSO), were first diluted to the highest concentration (100 mg/mL) to be tested, and 200 µL of the essential oils stock solutions were serial twofold diluted in a decreasing concentration range in the 96 well-plate. All 96-well plates were prepared by dispensing into each well 195 µL of the correspondent broth and 5 µL of the bacterial/fungal inocula. The last well containing 195 µL of nutrient broth without essential oil and 5 µL of the inocula on each strip was used as the negative control. The final volume in each well was 200 µL. The plates were incubated at 37 °C for 18–24 h.

The MIC value was determined as the lowest concentration of the sample that did not permit any visible growth of the tested microorganism after incubation. The MBC/MFC values were interpreted as the highest dilution (lowest concentration) of the sample, which showed clear fluid with no development of turbidity and without visible growth. All tests were performed in triplicate.

### 4.4. Anti-Quorum Sensing Activities of the Essential Oils

The anti-quorum sensing activities of the tested essential oils and their main component 1,8-cineole were tested by using three reporter strains *Pseudomonas aeruginosa* PAO1 and the mutant strain *Chromobacterium violaceum* CV026. The *P. aeruginosa* PAO1 strain was incubated at 37 °C for 24 h. While, the mini-Tn5 mutant *C. violaceum* 026 strains that produces violacein pigment only in the presence of exogenous C6-AHL was grown in Luria-Bertani broth medium at 32 °C.

#### 4.4.1. Effect on *P. aeruginosa* PAO1 Virulence Factors

The following QS-controlled virulence factors in *P. aeruginosa* PAO1 (swarming, elastolytic, and proteolytic activities) were tested as previously described [53,54].

The effect of the tested oils and 1,8-cineole on the swarming activity in *P. aeruginosa* PAO1 strain was performed in Luria Bertani semisolid medium (Biorad, France) prepared at 0.5% agar and supplemented with different oils concentrations ranging from 10 to 400 µg/mL. The Petri dishes plates inoculated with *P. aeruginosa* PAO1 were incubated for 24 h at 37 °C and the extent of swarming was determined by measuring the swarming diameter using a 1 cm flat ruler.

For elastolytic activity, *P. aeruginosa* PAO1 was grown in LB broth medium supplemented with different concentrations of essential oils and 1,8-cineole and incubated for 16 h at 37 °C. Then, 100 µL of the culture supernatant was added to 900 mL elastin congo red (ECR, Sigma-Aldrich S.r.l. Milan, Italy) buffer (100 mM Tris, 1 mM CaCl_2_, pH 7.5) containing 20 mg ECR (Sigma-Aldrich S.r.l. Milan, Italy). The eppendorf tubes were incubated for 3 h at 37 °C. The insoluble ECR was removed by centrifugation and the absorbance of the supernatant was estimated spectrophotometrically at OD_495_ (UV-1800, Shimadzu, Japan).

For proteolytic activity, 100 µL from the culture supernatant, obtained as mentioned above, was added to 900 mL ECR buffer containing 3 mg azocasein (Sigma-Aldrich S.r.l. Milan, Italy) and incubated for 30 min at 37 °C. Then, 100 µL of trichloroacetic acid (10%, *v*/*v*) was added to all reaction tubes. After 30 min, the tubes were centrifuged and absorbance of the supernatant was measured by reading spectrophotometrically at OD_440_ (UV-1800, Shimadzu, Kyoto, Japan).

#### 4.4.2. Effect on Violacein Production

Inhibition of violacein production in the presence of tested essential oils and their major component (1,8-cineole) was tested according the method described by McClean and colleagues [55]. For this, two microlitres of essential oil or 1,8-cineole (2 mg/disc) were loaded to the sterile disks and placed on the surface of CV026 inoculated LB agar plates supplemented with 50 µL of 1 µg/mL of C6-HSL. The plates were incubated upright at 32 °C for 24 h and zone of inhibition of QS was detected by the presence of colourless but viable cells around the disks and the zone of growth inhibition was also recorded.

### 4.5. Phytotoxic Activity of the Essential Oils

The phytotoxic activity was evaluated on germination and root elongation of three different plant species: *R. sativus* cv “Saxa” (radish), *L. sativum* (garden cress) and *L. sativa* (lettuce). These seeds are well known from the histological point of view and usually they are used in assays of phytotoxicity because of their easy germinability. The seeds of *R. sativus* and *L. sativum* were purchased from Blumen group srl (Piacenza, Italy), the seeds of garden cress from Euroselect (Bari). The seeds were surface sterilized in 95% ethanol for 15 s and sown in Petri dishes (Ø = 90 mm), containing five layers of Whatman filter paper, impregnated with distilled water (7 mL, control) or the tested solution of the essential oil (7 mL), at the different doses. The germination conditions were 20 ± 1 °C, with natural photoperiod. The essential oils, in water-acetone mixture (99.5:0.5), were assayed at the doses of 2.5, 1.25, 0.625, 0.25, 0.125 and 0.062 µg/mL Controls performed with water-acetone mixture alone showed no appreciable differences in comparison to controls in water alone. Seed germination was observed directly in Petri dishes every 24 h. A seed was considered germinated when the protrusion of the root became evident [56]. After 120 h (on the fifth day), the effects on radicle elongation were measured in cm. Each determination was repeated three times, using Petri dishes containing 10 seeds each. Data are expressed as the mean ± SD for both germination and radicle elongation [57].

### 4.6. Statistical Analysis

All data were expressed as Means ± Standard Deviation (S.D.). Each analysis was performed using the SPSS 16.0 statistics package for Windows (SPSS Inc., Chicago, IL, USA). The differences in mean were calculated using the Duncan’s multiple range tests for means with 95% confidence limit (*p* = 0.05). A principal component analysis (PCA) and cluster analysis (CA) were performed in order to discriminate between the three cardamom varieties on the basis of their essential oil composition. All analyses were performed by the “Statistica v 5.1” software (Statsoft. Inc., EEUU).

## Figures and Tables

**Table 1:** Essential oil composition (%) of different species of cardamoms studied.

Constituents	Ki *	Ki **	Essential Oils (%)		
			*E. cardamomum*	*A. corriroma*	*A. subulatum*
α-thujene	933	1035	0.1 ± 0.00	0.5 ± 0.1	-
α-pinene	941	1032	0.7 ± 0.1	1.4 ± 0.2	1.3 ± 0.2
sabinene	977	1132	1.5 ± 0.2	1.2 ± 0.2	-
β-pinene	982	1118	0.2 ± 0.1	4.6 ± 0.3	0.7 ± 0.1
6-methyl-5-hepten-2-one	987	1319	-	-	0.2 ± 0.1
myrcene	993	1174	1.2 ± 0.1	0.2 ± 0.1	0.4 ± 0.1
α-phellandrene	1006	1176	-	0.1 ± 0.0	1.6 ± 0.1
α-terpinene	1020	1188	-	0.4 ± 0.1	-
*p*-cymene	1028	1280	1.1 ± 0.2	4.5 ± 0.3	2.6 ± 0.3
limonene	1032	1203	2.5 ± 0.2	5.4 ± 0.4	2.4 ± 0.5
1.8-cineole	1034	1213	55.4 ± 1.5	51.8 ± 1.3	41.7 ± 1.6
γ-terpinene	1063	1255	0.1 ± 0.1	0.9 ± 0.1	0.3 ± 0.1
*cis*-sabinene hydrate	1070	1556	-	0.1 ± 0.1	-
*cis*-linalool oxide (furanoid)	1076	1450	0.1 ± 0.0	-	-
terpinolene	1090	1265	0.2 ± 0.1	0.4 ± 0.1	-
2-hexyl furan	1094		-	-	0.3 ± 0.1
linalool	1101	1553	0.9 ± 0.1	0.6 ± 0.1	3.0 ± 0.2
*cis*-*p*-menth-2-en-1-ol	1123	1230	0.1 ± 0.0	0.3 ± 0.1	-
*trans*-*p*-menth-2-en-1-ol	1142		-	0.4 ± 0.1	-
sabina ketone	1158	1652	-	0.1 ± 0.	-
δ-terpineol	1172		-	0.2±0.0	0.6 ± 0.1
4-terpineol	1179	1611	3.3 ± 0.3	10.4 ± 0.8	1.7 ± 0.2
*p*-cymen-8-ol	1185	1864	-	0.1 ± 0.1	-
α-terpineol	1191	1706	3.1 ± 0.3	4.9 ± 0.5	5.4 ± 0.4
*trans*-piperitol	1206		-	0.1 ± 0.1	-
neral	1242	1656	-	-	0.9 ± 0.2
carvone	1244	1752	-	0.2 ± 0.1	-
geraniol	1256	1857	-	1.9 ± 0.3	12.5 ± 0.8
linalyl acetate	1259	1565	0.4 ± 0.1	-	-
(*E*)-2-decenal	1263		-	-	0.7 ± 0.1
geranial	1271	1267	-	0.2 ± 0.0	3.1 ± 0.1
2-phenyl-2-butenal	1285		-	-	2.9 ± 0.1
Thymol	1292	2198	-	-	0.3 ± 0.1
*cis*-2.3-pinanediol	1315		-	-	0.7 ± 0.1
methyl geranate	1325		0.2 ± 0.0	-	-
myrtenyl acetate	1327	1698	-	0.1 ± 0.0	-
*exo*-2-hydroxycineol acetate	1345		-	0.3 ± 0.1	-
α-terpinyl acetate	1352	1709	28.6 ± 1.1	3.6 ± 0.5	-
α-copaene	1377	1497	-	0.1 ± 0.0	-
geranyl acetate	1385	1765	0.2 ± 0.1	1.2 ± 0.3	6.0 ± 0.7
(*E*)-2-decenyl acetate	1407		-	-	1.1 ± 0.1
β-caryophyllene	1419	1612	-	0.4±0.1	
γ-muurolene	1478	1704	-	-	0.3 ± 0.1
δ-cadinene	1524	1773	-	-	0.3 ± 0.1
elemol	1550		-	-	0.2 ± 0.0
(*E*)-nerolidol	1565	2050	-	1.3±0.2	1.1 ± 0.1
caryophyllene oxide	1582	2008	-	0.3±0.1	-
Oxygenated monoterpenes			63.0 ± 0.3	71.4 ± 0.3	51.0 ± 0.4
Monoterpene hydrocarbons			36.9 ± 0.2	24.7 ± 0.2	34.2 ± 0.3
Sesquiterpene hydrocarbons			0.0	0.5 ± 0.1	0.6 ± 0.1
Oxygenated sesquiterpenes			0.0	1.6 ± 0.2	1.3 ± 0.1
Others			0.0	0.0	5.2 ± 0.1
Total identified			99.9	98.2	92.3

* Kovats retention index determined relative to the tR of a series of n-alkanes (C_10_–C_35_) on a HP-5 MS column; ** Kovats retention index determined relative to the tR of a series of n-alkanes (C_10_–C_35_) on HP Innowax.

**Table 2:** Antifungal activity of cardamom essential oils as compared to the drug agent (Amphotericin B) as determined by the disc diffusion and microdilution assays.

Microorganisms	*Green cardamom*			*Ethiopian cardamom*			*Black cardamom*			Amphotericin B (10 mg/mL)
	ZI * ± SD **	MIC ***	MFC ****	ZI * ±SD **	MIC ***	MFC ****	ZI ± SD	MIC	MFC	
*Candida tropicalis* 06–085	15.33 ± 0.57 ^i^	0.048	6.25	34.33 ± 0.57 ^a^	0.048	0.19	6.00 ± 0.00 ^l^	0.048	3.125	-
*Candida parapsilosis* ATCC 22019	21.67 ± 0.57 ^cd^	0.048	6.25	24.33 ± 0.57 ^f^	0.19	0.78	6.00 ± 0.00 ^l^	0.097	6.25	10.33 ± 0.57
*Candida krusei* ATCC 6258	14.33 ± 0.57 ^ij^	0.048	12.5	15.33 ± 0.57 ^jkl^	0.097	1.75	6.00 ± 0.00 ^l^	0.048	6.25	12.00 ± 0.00
*Candida glabrata* ATCC 900030	16.33 ± 0.57 ^h^	0.097	12.5	31.00 ± 1.00 ^bc^	0.097	078	40.33 ± 0.57 ^b^	0.048	6.25	14.33 ± 0.57
*Candida guilliermondi* 06–018	16.67 ± 0.57 ^gh^	0.048	6.25	30.33 ± 0.57 ^cd^	0.097	0.78	41.00 ± 0.00^b^	0.097	3.125	-
*Candida albicans* ATCC 2019	15.33 ± 0.57 ^i^	0.097	6.25	12.67 ± 0.57 ^nop^	0.097	0.39	12.00 ± 0.00 ^h^	0.048	3.125	14.66 ± 0.57
*Saccharomyces cerevisiae* 11–161	18.67 ± 0.57 ^f^	0.097	6.25	21.67 ± 0.57 ^g^	0.097	0.39	40.33 ± 0.57 ^b^	0.048	6.25	-

* ZI: Inhibition zone in diameter (mm ± SD) around the discs impregnated with 10 mg/disk of essential oil including disc diameter. ** SD: standard deviation; (a–q): Means followed by the same letters are not significantly different at *p* = 0.05 based on Duncan’s multiple range tests. *** MIC: Minimum Inhibitory Concentration (mg/mL). **** MFC: Minimum Fungicidal Concentration (mg/mL).

**Table 3:** Antibacterial activity of cardamom essential oils as compared to the drug agent (Ampicillin) determined by the disc diffusion and microdilution assays.

Microorganisms	*Green cardamom*			*Ethiopian cardamom*			*Black cardamom*			Ampicillin (10 mg/mL)
	ZI * ± SD **	MIC ***	MBC ****	ZI * ± SD **	MIC ***	MBC ****	ZI * ± SD **	MIC ***	MBC ****	
*Aerococcus viridans*	20.33 ± 0.57 ^e^	0.048	6.25	25.67 ± 0.57 ^e^	0.048	0.39	35.00 ± 0.00 ^c^	0.048	3.125	14.67 ± 0.57
*Bacillus cereus*	11.67 ± 0.57 ^n^	0.048	6.25	16.33 ± 0.57 ^ij^	0.048	3.125	35.67 ± 0.57 ^c^	0.048	1.56	-
*Bacillus subtilis* ATCC 6633	12.67 ± 0.57 ^klmn^	0.048	12.5	14.67 ± 0.57 ^klm^	0.048	3.125	7.00 ± 0.00 ^k^	0.048	0.78	11.33 ± 0.57
*Enterococcus faecalis* ATCC 29212	21.00 ± 1.00 ^de^	0.048	6.25	12.00 ± 0.00 ^opq^	0.048	12.5	13.67 ± 0.57 ^g^	0.048	6.25	13.67 ± 0.57
*Listeria monocytogenes* ATCC 19115	17.33 ± 0.57 ^g^	0.048	12.5	14.33 ± 0.57 ^lm^	0.19	6.25	6.00 ± 0.00 ^l^	0.048	1.56	-
*Micrococcus luteus* NCIMB 8166	41.33 ± 1.15 ^a^	0.048	6.25	29.33 ± 0.57 ^d^	0.048	6.25	7.67 ± 0.57 ^jk^	0.048	3.125	30.33 ± 0.57
*Staphylococcus aureus* MR (B2)	13.67 ± 0.57 ^jk^	0.048	12.5	17.00 ± 1.00 ^i^	0.048	12.5	6.00 ± 0.00 ^l^	0.048	6.25	16.33 ± 0.57
*Staphylococcus aureus* ATCC 6816	32.67 ± 0.57 ^b^	0.048	6.25	32.00 ± 0.00 ^b^	0.097	12.5	19.67 ± 0.57 ^f^	0.048	3.125	24.33 ± 0.57
*Staphylococcus epidermidis* ATCC 12228	11.67 ± 0.57 ^n^	0.048	6.25	13.67 ± 0.57 ^mn^	0.097	6.25	43.00 ± 1.00 ^a^	0.097	1.56	12.33 ± 0.57
*Escherichia coli*	12.00 ± 0.00 ^mn^	0.097	6.25	13.00 ± 1.00 ^no^	0.048	3.125	6.00 ± 0.00 ^l^	0.048	1.56	27.33 ± 0.57
*Escherichia coli* ATCC 25922	15.33 ± 0.57 ^i^	0.048	12.5	15.33 ± 0.57 ^jkl^	0.048	12.5	6.00 ± 0.00 ^l^	0.048	0.78	11.67 ± 0.57
*Klebsiella pneumoniae*	10.33 ± 0.57 ^o^	0.048	12.5	12.00 ± 0.00 ^opq^	0.097	3.125	8.33 ± 0.57 ^j^	0.097	6.25	17.33 ± 0.57
*Pseudomonas aeruginosa* ATCC 27853	6.00 ± 0.00 ^r^	0.048	12.5	11.33 ± 0.57 ^q^	0.048	3.125	10.33 ± 0.57 ^i^	0.048	3.125	22.67 ± 0.57
*Proteus mirabils*	8.00 ± 0.00 ^p^	0.048	25	11.67 ± 0.57 ^pq^	0.048	6.25	13.67 ± 0.57 ^g^	0.048	12.5	25.67 ± 0.57
*Shewanella putrefaciens*	13.00 ± 0.00 ^klm^	0.097	12.5	15.33 ± 0.57 ^jkl^	0.19	6.25	30.33 ± 0.57 ^d^	0.048	3.125	7.00 ± 0.00
*Salmonella typhimirium* ATCC 14028	10.00 ± 0.00 ^o^	0.048	12.5	9.33 ± 0.57 ^r^	0.048	3.125	7.67 ± 0.57 ^jk^	0.048	0.78	17.67 ± 1.15
*Shigella flexenerii* ATCC 12022	22.33 ± 0.57 ^c^	0.048	6.25	19.67 ± 0.57 ^h^	0.048	6.25	14.33 ± 0.57 ^g^	0.048	0.39	10.67 ± 0.57
*Vibrio alginolyticus* ATCC 17749	20.67 ± 0.57 ^de^	0.048	3.125	16.33 ± 0.57 ^ij^	0.097	25	7.00 ± 0.00 ^k^	0.048	1.56	-
*Vibrio alginolyticus* ATCC 33787	12.33 ± 0.57 ^lmn^	0.048	12.5	15.33 ± 0.57 ^jkl^	0.097	25	26.67 ± 0.57 ^e^	0.048	6.25	13.33 ± 0.57
*Vibrio cholerae* ATCC 9459	7.00 ± 0.00 ^q^	0.097	12.5	14.33 ± 0.57 ^lm^	0.048	25	6.00 ± 0.00 ^l^	0.048	12.5	7.00 ± 0.00
*Vibrio parahaemolyticus* ATCC 17802	20.67 ± 0.57 ^de^	0.097	12.5	14.33 ± 0.57 ^lm^	0.048	12.5	7.00 ± 0.00 ^k^	0.048	1.56	13.33 ± 0.57
*Vibrio parahaemolyticus* ATCC 43996	21.67 ± 0.57 ^cd^	0.048	12.5	14.33 ± 0.57 ^lm^	0.048	25	6.00 ± 0.00 ^l^	0.048	6.25	12.00 ± 0.00
*Vibrio vulnificus* ATCC 27562	13.00 ± 0.00 ^klm^	0.048	12.5	15.67 ± 0.57 ^jk^	0.097	12.5	29.67 ± 0.57 ^d^	0.097	6.25	30.33 ± 0.57
*Vibrio vulnificus* ATCC 33149	13.33 ± 0.57 ^o^	0.048	12.5	13.00 ± 1.00 ^no^	0.048	6.25	6.00 ± 0.00 ^l^	0.048	6.25	12.33 ± 0.57
*Serratia marcescens*	14.33 ± 0.57 ^ij^	0.048	12.5	12.33 ± 0.57 ^opq^	0.048	3.125	8.33 ± 0.57 ^j^	0.048	3.125	13.67 ± 0.57

* ZI: Inhibition zone in diameter (mm ± SD) around the discs impregnated with 10 mg/disk of essential oil including disc diameter. ** SD: standard deviation; (a–q): Means followed by the same letters are not significantly different at *p* = 0.05 based on Duncan’s multiple range tests. *** MIC: Minimum Inhibitory Concentration (mg/mL). **** MBC: Minimum Bactericidal Concentration (mg/mL).

**Table 4:** Effect of the three essential oils tested and 1,8-cineole at different concentrations (on swarming motility in PAO1 tested on LB semisolid medium.

Concentration (µg/mL)	Essential Oils			Main Component 1,8-Cineole
	*Green cardamom*	*Black cardamom*	*Ethiopian cardamom*	
	M ± SD *	M ± SD *	M ± SD *	M ± SD *
Control	54.00 ± 1.00	54.00 ± 1.00	54.00 ± 1.00	54.00 ± 1.00
10	22.00 ± 1.00	52.33 ± 0.58	53.76 ± 0.58	17.33 ± 1.15
50	17.67 ± 0.58	50.67 ± 1.15	53.00 ± 0.00	15.00 ± 0.00
100	13.67 ± 0.58	46.76 ± 1.15	32.67 ± 0.58	13.67 ± 0.58
200	11.13 ± 1.00	35.76 ± 0.58	13.33 ± 1.15	12.33 ± 0.58
400	8.33 ± 0.58	33.00 ± 2.65	8.33 ± 0.58	10.33 ± 0.58

* M ± SD: Mean diameter expressed in mm ± standard deviation.

**Table 5:** Phytotoxic activity of the three essential oils of *A. corriroma*, *A. subulatum*, and *E. cardamomum* against germination of *R. sativus*, *L. sativum*, and *L. sativa* seeds. Data expressed in number of seeds germinated ± SD after 120 h of sowing. ** *p* < 0.01; * *p* < 0.05.

***Raphanus sativus* Germinated Seeds**			
**Doses (µg/mL)**	***A. corriroma***	***A. subulatum***	***E. cardamomum***
Control	5.70 ± 1.50	5.00 ± 1.70	7.30 ± 1.20
0.062	7.30 ± 1.20	4.70 ± 1.20	4.00 ± 0.00
0.125	5.30 ± 2.30	7.00 ± 1.00	4.70 ± 2.50
0.250	4.33 ± 2.08	6.00 ± 1.00	5.67 ± 1.53
0.625	5.00 ± 1.00 *	6.33 ± 2.08	5.33 ± 0.58
1.25	5.00 ± 1.00 *	5.67 ± 1.53	5.67 ± 2.89
2.5	3.67 ± 0.58 **	5.00 ± 1.73	6.67 ± 2.52
***Lactuca sativa* Germinated Seeds**			
**Doses (µg/mL)**	***A. corriroma***	***A. subulatum***	***E. cardamomum***
Control	8.70 ± 0.60	9.30 ± 1.20	6.70 ± 0.60
0.062	8.30 ± 0.60	8.70 ± 1.50	8.00 ± 1.00
0.125	8.70 ± 0.60	8.70 ± 0.60	9.70 ± 0.60
0.250	8.00 ± 1.00	8.70 ± 1.50	9.00 ± 1.00
0.625	9.00 ± 1.00	8.70 ± 1.20	8.30 ± 2.10
1.25	9.00 ± 0.00	8.70 ± 1.50	8.30 ± 0.60
2.5	8.00 ± 1.00	8.30 ± 1.50	8.70 ± 0.60
***Lepidium sativum* Germinated Seeds**			
**Doses (µg/mL)**	***A. corriroma***	***A. subulatum***	***E. cardamomum***
Control	3.30 ± 0.60	6.3 ± 1.50	4.30 ± 1.20
0.062	4.70 ± 2.50	6.00 ± 1.70	5.30 ± 1.20
0.125	2.30 ± 1.50	5.30 ± 1.50	6.00 ± 2.60
0.250	3.67 ± 2.50	4.67 ± 0.60	6.00 ± 2.00
0.625	2.30 ± 0.60	6.70 ± 1.20	4.00 ± 3.50
1.25	4.00 ± 2.00	3.70 ± 2.10	3.30 ± 2.10
2.5	3.30 ± 1.50	4.70 ± 2.30	4.00 ± 1.70
